# Erythrocyte miRNA-92a-3p interactions with PfEMP1 as determinants of clinical malaria

**DOI:** 10.1007/s10142-023-01028-w

**Published:** 2023-03-20

**Authors:** Sowmya R. Prabhu, Akshay Pramod Ware, Shashikiran Umakanth, Manjunath Hande, Chakrapani Mahabala, Abdul Vahab Saadi, Kapaettu Satyamoorthy

**Affiliations:** 1 Department of Biotechnology, Manipal School of Life Sciences, Manipal Academy of Higher Education, Manipal, Karnataka 576104 India; 2 Department of Bioinformatics, Manipal School of Life Sciences, Manipal Academy of Higher Education, Manipal, Karnataka 576104 India; 3 Department of Medicine, Dr. TMA Pai Hospital, Manipal Academy of Higher Education, Manipal, Karnataka 576104 India; 4 Department of Medicine, Kasturba Medical College, Manipal Academy of Higher Education, Manipal, Karnataka 576104 India; 5 Department of Medicine, Kasturba Medical College, Mangalore, Manipal Academy of Higher Education, Manipal, Karnataka 576104 India; 6 Department of Cell and Molecular Biology, Manipal School of Life Sciences, Manipal Academy of Higher Education, Manipal, Karnataka 576104 India

**Keywords:** Malaria, *Plasmodium*, Erythrocyte, miR-92a-3p, Exoribonuclease

## Abstract

Based on the recently added high throughput analysis data on small noncoding RNAs in modulating disease pathophysiology of malaria, we performed an integrative computational analysis for exploring the role of human-host erythrocytic microRNAs (miRNAs) and their influence on parasite survival and host homeostasis. An in silico analysis was performed on transcriptomic datasets accessed from PlasmoDB and Gene Expression Omnibus (GEO) repositories analyzed using miRanda, miRTarBase, mirDIP, and miRDB to identify the candidate miRNAs that were further subjected to network analysis using MCODE and DAVID. This was followed by immune infiltration analysis and screening for RNA degradation mechanisms. Seven erythrocytic miRNAs, miR-451a, miR-92a-3p, miR-16-5p, miR-142-3p, miR-15b-5p, miR-19b-3p, and miR-223-3p showed favourable interactions with parasite genes expressed during blood stage infection. The miR-92a-3p that targeted the virulence gene PfEMP1 showed drastic reduction during infection. Performing pathway analysis for the human-host gene targets for the miRNA identified *TOB1*, *TOB2*, *CNOT4*, and *XRN1* genes that are associated to RNA degradation processes, with the exoribonuclease *XRN1*, highly enriched in the malarial samples. On evaluating the role of exoribonucleases in miRNA degradation further, the pattern of *Plasmodium falciparum*_XRN1 showed increased levels during infection thus suggesting a defensive role for parasite survival. This study identifies miR-92a-3p, a member of C13orf25/ miR-17-92 cluster, as a novel miRNA inhibitor of the crucial parasite genes responsible for symptomatic malaria. Evidence for a plausible link to chromosome 13q31.3 loci controlling the epigenetic disease regulation is also suggested.

**Supplementary Information:**

The online version contains supplementary material available at 10.1007/s10142-023-01028-w.

## Introduction

Malaria, which is classified as one of the lethal vector-borne infectious disease by the World Health Organization (WHO), exhibits a high morbidity and mortality worldwide affecting half a million lives annually. Caused by five species of the protozoan *Plasmodium* parasite, *P. falciparum*, and *P. vivax* contribute to sizable infections often leading to death. In humans, the parasite has an early developmental stage in the liver following which the egressed merozoites infect the erythrocytes for further proliferation (Votýpka et al. 2016; Sato 2021). The erythrocyte-stage is clinically symptomatic with an exponential increase in the number of the parasites accounting for the high parasitemia (Kariuki and Williams 2020). In endemic areas for malaria, the prevalence of several host genetic variants that bring about resistance to malaria have been discovered. Population genetic studies have found rare variants associated with genetic diseases augmenting resistance to malaria have increased in their geographic distribution and these include a and b thalassemia (*HBA1*, *HBA2*, *HBB*), sickle-cell anaemia (*HBB*), glucose-6-phosphate dehydrogenase (*G6PD*) deficiency, human leukocyte antigen (*HLA*), and *ABO*. This being influenced by, but not limited to the resistance selection along with gene flow, genetic drift, mutation, and interaction between the factors (Hedrick 2011). Heterozygote advantage for sickle cell haemoglobin (HbAS) demonstrating resistance against *P. falciparum* infections is due to changes in the morphology of the cells, phagocytosis of infected sickle cells, and also translocation of human microRNAs (miRNAs/miR) into the parasite (LaMonte et al. 2012; Archer et al. 2018). Host immune response, parasite species and their variants, host genetics, and treatment strategies all contribute towards the outcome of the infection (Huang et al. 2018).

Dysregulation of miRNAs in several diseases have helped researchers to evaluate their potential role in pathophysiology. Circulating miRNAs have been implicated to predict the severity of the disease with high specificity and sensitivity in malaria patients (Martin-Alonso et al. 2018). Tissue-specific miRNAs are disseminated into the blood stream when the liver or other tissues are infected by the malaria parasites. In early, severe and/or cerebral malaria, the association of miRNAs such as miRNA-16, miRNA-155, miRNA-150, miRNA-223, and miRNA-451 in the dysregulation of expression of genes involved in immune regulation signifies the role of these miRNAs as biological indicators of malaria infection (Rangel et al. 2019). Experiments have demonstrated that the host miRNAs appear to target the parasite mRNAs, thereby inhibiting their translation and negatively regulating the parasite growth (LaMonte et al. 2012).

The life cycle of *Plasmodium* within their human host is well known, and the interventional strategies such as chemotherapy (including treatments with artemisinin-based combination therapies, impeding the dormant hypnozoite stages) and vaccines e.g., RTS, S/AS01 (Mosquirix), PfGAP3KO have been employed to disrupt the progression of parasites (Campo et al. 2015; van der Pluijm et al. 2020; WHO 2021; Murphy et al. 2022). Augmenting this, understanding the interactions between the pathogens and the host tissues may expand our knowledge for development of strategies that could target these interactions to control the *Plasmodium* effects in humans (Simões et al. 2018). *Plasmodium* genome comprises of 14 chromosomes and encodes approximately 5300 genes (Gardner et al. 2002). Evaluating the role of parasitic candidate genes involved in various stages of infection and their interaction with human host factors may also provide an effective strategy for therapeutic intervention. Host-mediated epigenetic regulation of plasmodial genes has been demonstrated by investigators such as LaMonte et al., 2012. Other studies have associated the role of miRNAs in modifying the clinical phenotypes serving as useful biomarkers of malaria (LaMonte et al. 2012; Mantel et al. 2016; Chakrabarti et al. 2020; Gupta et al. 2021a). Currently, we evaluate the spectrum of human erythrocyte miRNAs expressed during malarial infection for validation of their putative role in modulating parasite genes critical for the erythrocytic phase that has potential to influence disease outcome.

## Materials and methods

### Acquisition of *Plasmodium falciparum* transcripts and erythrocytic miRNAs

**Fig. 1:**
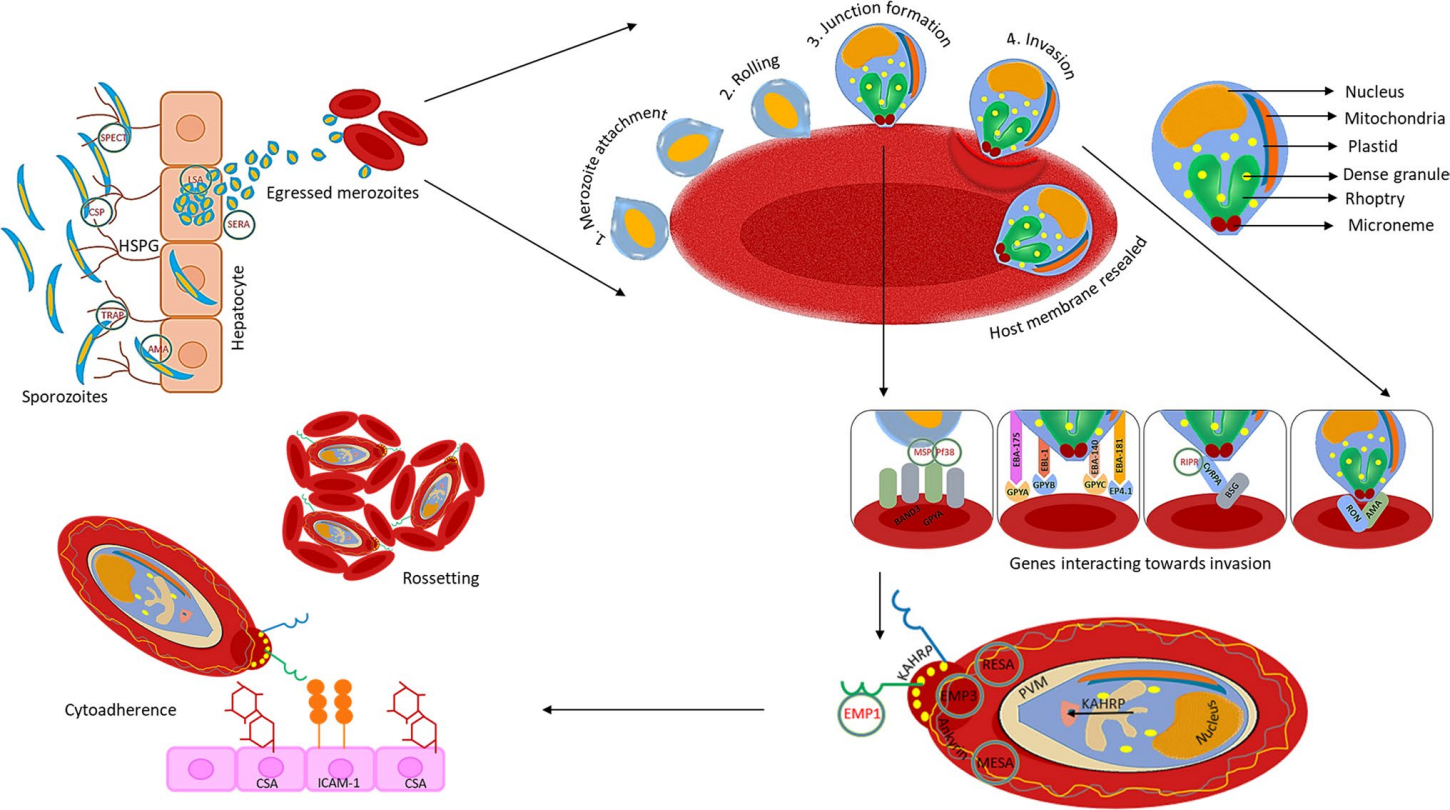
Summary of the involvement of parasite genes in invasion into and egress from the liver and erythrocyte during malaria (Abbreviations: AMA, apical membrane antigen; BSG, basigin; CSA, chondroitin sulphate A; CSP, circumsporozoite protein; CyRPA, cysteine-rich protective antigen; EBA, erythrocyte binding antigen; EBL, erythrocyte binding like; EMP, erythrocyte membrane protein; GPY, glycophorin; HSPG, heparan sulfate proteoglycan; ICAM, intercellular adhesion molecule 1; KAHRP, knob-associated histidine-rich protein; LSA, liver stage antigen; MESA, mature-parasite-infected erythrocyte surface antigen; MSP, merozoite surface protein; PVM, parasitophorous vacuolar membrane; RESA, ring-infected erythrocyte surface antigen; RIPR, PfRh5-interacting protein; RON, rhoptry neck protein; SERA, serine repeat antigen; SPECT, sporozoite microneme protein essential for cell traversal; TRAP, thrombospondin-related anonymous protein)

Our analysis involved identification of *Plasmodium* genes known for invasion into and egress from the erythrocytes (shown schematically in Fig. 1) for which transcripts of all reference sequences were retrieved from PlasmoDB (release 54) presented in Supplementary Table S1 (Aurrecoechea et al. 2009). The published data of LaMonte et al. 2012, Mantel et al. 2016, and Dieng et al. 2020 were used as the source data for all our analysis of host erythrocytic miRNAs and their expression profiles (LaMonte et al. 2012; Mantel et al. 2016; Dieng et al. 2020).

### Predicting effective host miRNA target sites in parasite mRNAs and their expression patterns

**Fig. 2:**
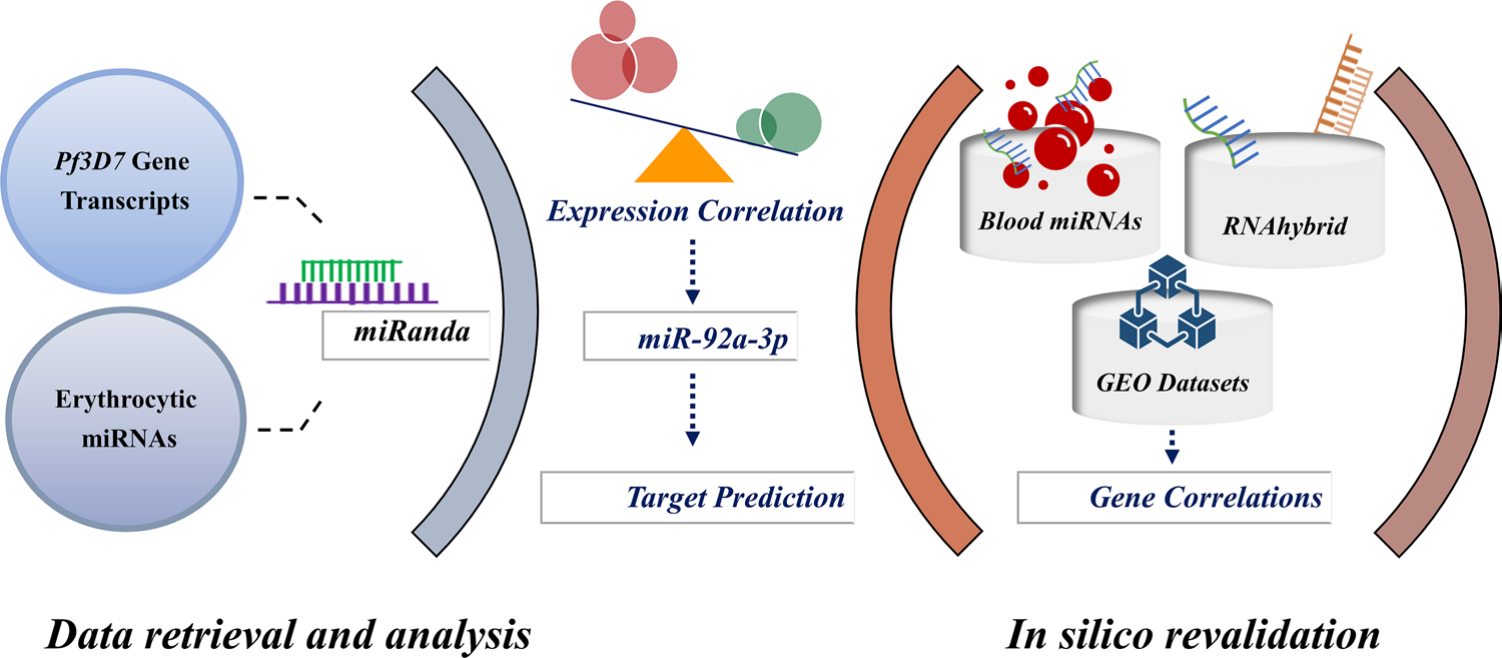
Design of the study. Erythrocytic miRNAs and *P. falciparum* gene transcripts were retrieved and analyzed for their potential interactions. The characteristic miRNA identified was then subjected to integrative in silico analysis and predicted for its human host targets. Blood miRs database, *RNAhybrid* and NCBI GEO were employed to revalidate the in silico findings

The parasite mRNA interactions for the *plasmodial* strains of *Pf3D7* with 175 host miRNAs were predicted by using miRanda (v1.9), an algorithm used for finding potential targets for miRNAs which sorts the interactions based on the score and energy (Enright et al. 2003; John et al. 2004). The interactions were sorted and selected considering stringent parameters with either a pairing score of >150 or an energy score of ≤15 (Riffo-Campos et al. 2016). Expression profiles of the interactions, i.e., RNA-Seq transcription data of the *Plasmodium* transcripts deposited by Otto et al. 2010 in PlasmoDB and erythrocyte miRNA expression by LaMonte et al. 2012, were also compared to explore their correlation patterns at different time points of infection (LaMonte et al. 2012; Otto et al. 2010). The experiment design is presented schematically in Fig. 2.

### Computational revalidation

The presence of erythrocytic miRNAs were examined using blood miRNA database and potential miRNA-mRNA interactions obtained from miRanda tool were revalidated using RNA*hybrid* tool (Rehmsmeier et al. 2004; Juzenas et al. 2017). Expression data was procured from GEO repositories (GSE144413, GSE144476, GSE50957, GSE52166) and differentially expressed miRNAs and gene transcripts in malaria patients were compared and analysed.

### Integrative analysis of the characteristic miRNA: miR-92a-3p

The human host gene-targets of miR-92a-3p were obtained from miRTarBase (Huang et al. 2020), mirDIP (Tokar et al. 2018), and miRDB (Chen et al. 2020) databases. The genes found to be common in these three databases were identified for further downstream analysis.

To predict the functional protein association networks among the target genes, the targets were mapped to the STRING v11.5 (Szklarczyk et al. 2019) with a medium confidence score ≥ 0.40. The Molecular Complex Detection (MCODE) plugin from Cytoscape (Bader and Hogue 2003) was utilized for distinguishing the module that best represents the clusters of target genes. Strict cut-off requirements were applied (degree cut-off = 2; node score cut-off = 0.2; k-core = 2; max depth = 100). The significant modules were used to generate and visualize networks, based on GO-enrichment analysis (GOEA) by using ToppGene (Chen et al. 2009). BioLayout Express3D tool (Theocharidis et al. 2009) was used for module visualization. Additionally, we performed the functional enrichment analysis for 289 target genes using DAVID platform (Sherman et al. 2022). The *p* value was calculated using the Benjamini–Hochberg method and ≤ 0.05 threshold was considered statistically significant, and expression levels of the genes involved in the RNA degradation pathways were further analyzed in the samples obtained from GEO studies (GSE50957, GSE52166).

### Immune infiltration analysis

Gene expression profiles (GSE144476, *n*=51) obtained during the symptomatic phase of infected individuals was used to estimate the quantity of distinct immune cell types by using CIBERSORT to obtain a matrix of 22 different types of immune cells (Newman et al. 2015). We generated the relative immune fraction score in the CIBERSORT exploratory analysis that estimates the proportion of each immune cell type, such that the total of all fractions is equal to 100%. The proportion of each cell type was computed and plotted as a bar graph using the *ggplot2*. Subsequently, the *corrplot* R package was used to generate a correlation heatmap to reveal the correlation of 15 immune cells.

### Structure, location, and regulation of miR-92a-3p

Domains of the PfEMP1 genes for the reference strain, *Pf3D7*, were curated from Uniprot database and analyzed for the miRNA-binding motifs (UniProt Consortium 2021). Subsequently, 460 popset isolate sequences for the gene were retrieved from PlasmoDB and explored for the miRNA-binding sites in the domains of PfEMP1; the domain-specific subtypes of which were obtained from the European Nucleotide Archive (ENA) repository (https://www.ebi.ac.uk/ena/browser/home). The local database of these sequences was then constructed and alignment for the miR-92a-3p binding region was performed using the standalone version of NCBI blast (Altschul et al. 1990). The alignments for the target sites were then visualized using MEGA11.0 (Kumar et al. 2016). The frequency of the miR-complementary sequences of various lengths with respect to their targeting domain subtypes were computed in the patient samples and mapped to their clinical phenotypes. The UCSC genome browser was used to locate the miRNA structure and position (Kent et al. 2002).

## Results

### Screening of putative miRNA targets

**Fig. 3:**
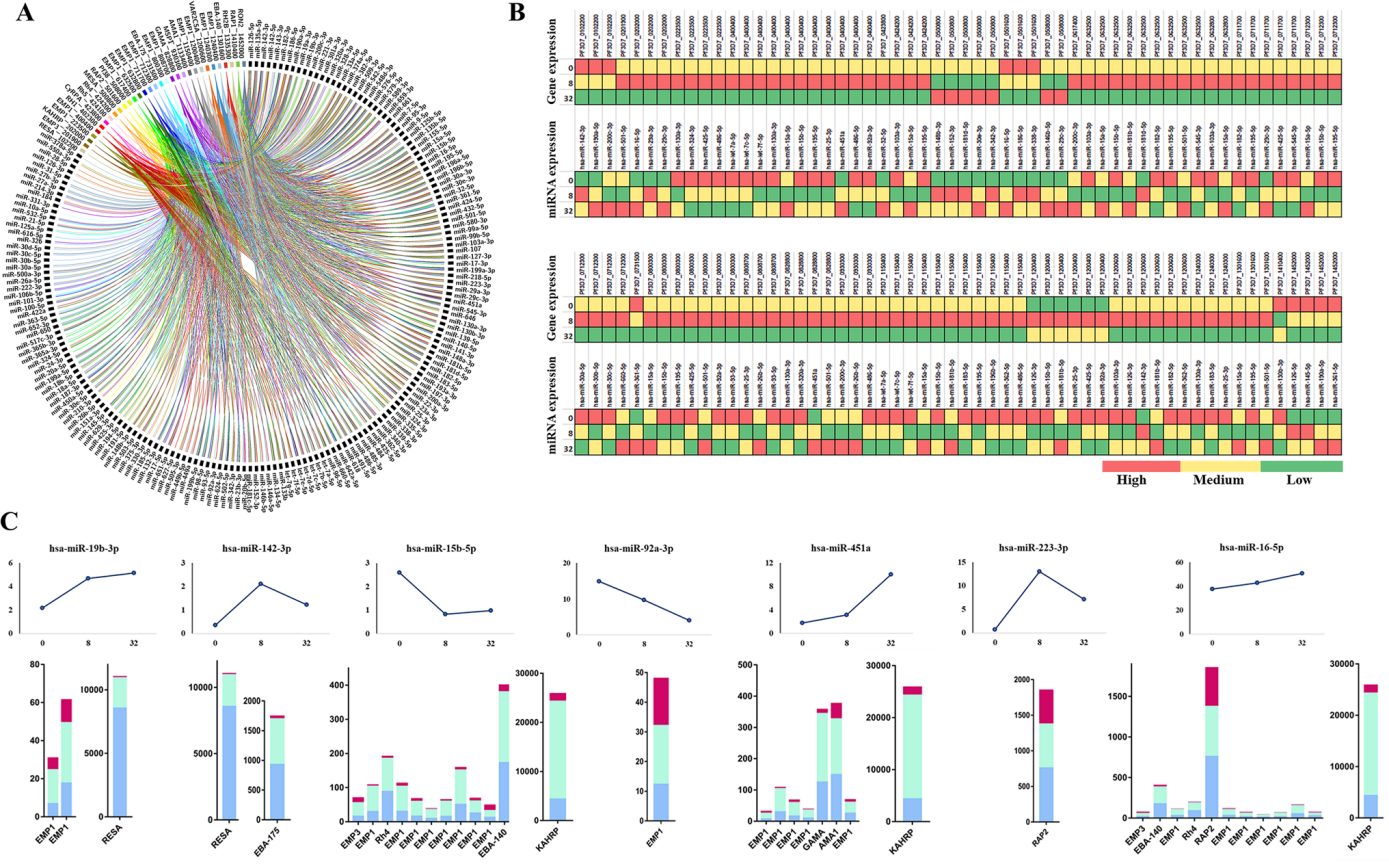
Interaction network and analysis of host erythrocytic miRNAs with its *Plasmodium* mRNA targets. **A** The miRNA-mRNA regulatory networks were visualized by using Circos tool. **B** Heatmap showing expression patterns of human host miRNAs and the parasite mRNA targets at 0h, 8h, and 32h post-*P. falciparum* infection. A color-coding system red, yellow, and green differentiates the high, medium, and low expression values, respectively. **C** miRNAs showing significant change in expression with their interacting genes (TPM) at 0h, 8h, and 32h of infection is depicted in the bar graph. Blue (0h), green (8h), and pink (32h) color denoting the *Plasmodium* gene expression profiles (bar graphs generated using GraphPad Prism 8.0 software, San Diego, CA, USA). The trendline graph demonstrates time (h) vs expression profiles (percentage) of the miRNAs

Target sites for the *Plasmodium* genes were identified for the miRNAs expressed in erythrocytes when the transcripts of selected genes were analysed with the miRanda target prediction tool. The Supplementary Table S2 lists the preferred miRNAs following the criteria for stringent selection parameters where 2105 target sites for *P. falciparum* transcripts for 175 of the miRNAs were identified. The miRNA-mRNA regulatory networks were visualized by using Circos (Fig. 3A). It is highly possible that if several miRNAs target a single gene, they might strongly suppress its expression (Peter 2010). Therefore, we investigated the number of miRNAs targeting a particular gene (Supplementary Table S3). The top five transcripts targeted by a majority of these miRNAs are presented in Table 1.

**Table 1:** Parasite genes that were identified to have multiple targets for different host miRNAs

No	PlasmoDB ID	Chromosome no.	Gene	No. of miRNAs targeting Pf3D7_EMP1
1	PF3D7_1150400	11	PfEMP1	86
2	PF3D7_0632500	06	PfEMP1	77
3	PF3D7_0400400	04	PfEMP1	74
4	PF3D7_1200600	12	PfEMP1	72
5	PF3D7_0800300	08	PfEMP1	68

### Comparing the contrasting expression profiles

Changes in the expression profiles of individual miRNAs in relation to their interacting gene expression patterns were analysed. Gene expressions at 0h, 8h, and 32h post-*Plasmodium* infections were assessed and compared with targeting miRNA expression from uninfected erythrocytes and at 8h and 32h post-infections, respectively (Supplementary Table S4). A color-coding system was used to differentiate the high, medium, and low expression values found as presented in the heatmap (Fig. 3B). The miRNAs not detected (ND) were excluded from the final analysis. Figure 3C presents the seven miRNAs (miR-451a, miR-92a-3p, miR-16-5p, miR-142-3p, miR-15b-5p, miR-19b-3p, and miR-223-3p) with significant changes in their expression during the course of infection, among which miR-451a, miR-15b-5p, and miR-16-5p were found to target multiple gene sequences. Out of the seven host miRNAs predicted to target the *Plasmodium* genes of interest, miR-92a-3p revealed contrasting outcomes as compared to that of other miRNAs shown in Fig. 3C. miR-92a-3p, being the primary miRNA both in terms of abundance and specificity to target the virulent gene, erythrocyte membrane protein (PfEMP1), was found to substantially reduce during the course of infection (highest levels observed at 0h and levels decrease at 32h of infection). With depletion in the miR-92a-3p levels from 0 to 8h, an elevated expression in the levels of PfEMP_1200400 was observed during the same time interval. These seven miRNAs and miR-92a-3p in general were considered for further downstream computational analysis.

### Conformity of the in silico outcomes

**Fig. 4:**
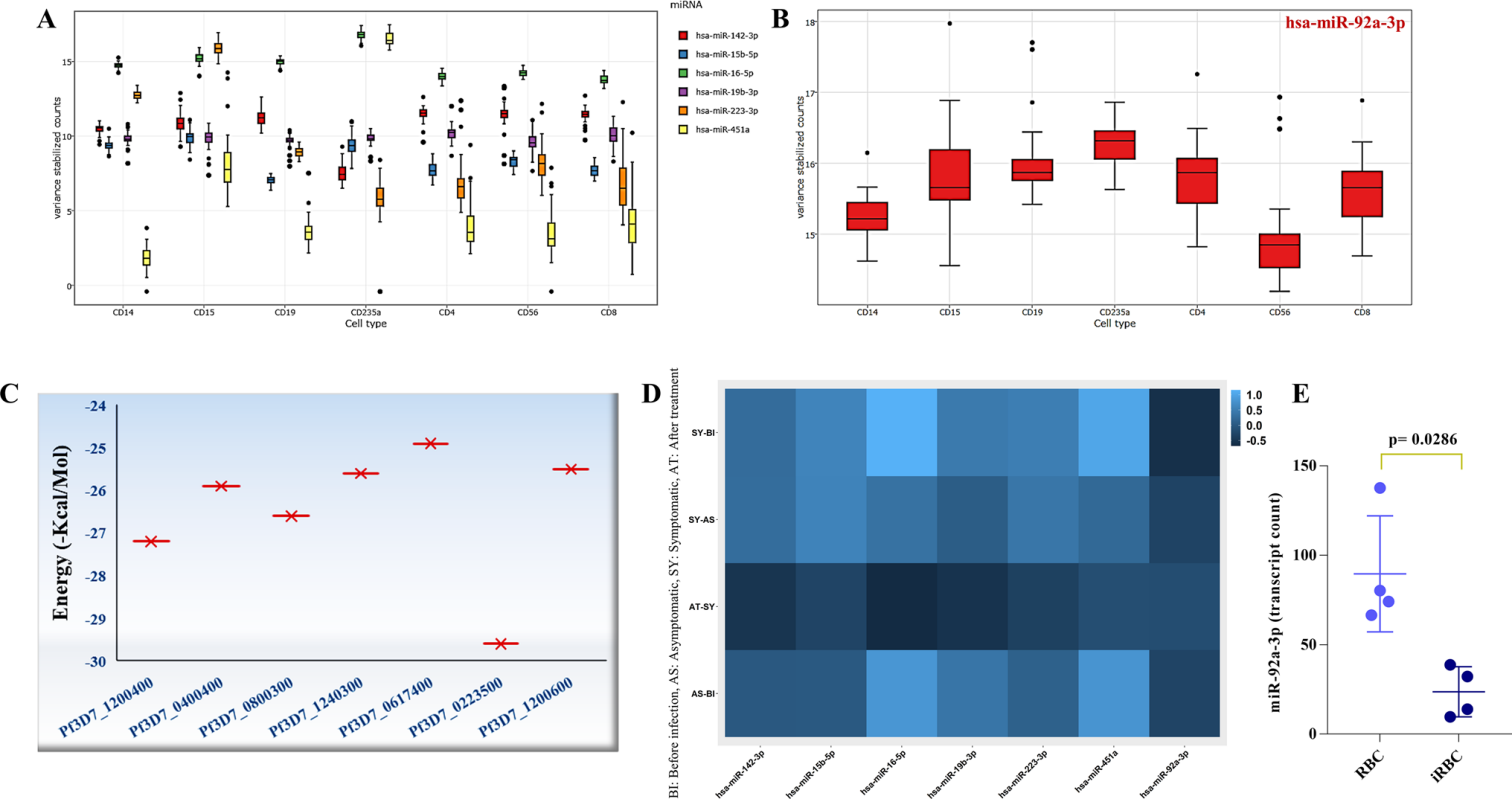
In silico revalidation. **A** Expression profiles of the six miRNAs (miR-19b-3p, miR-142-3p, miR-15b-5p, miR-451a, miR-16-5p, miR-223-3p) and (**B**) miR-92a-3p, in different types of blood cells—CD14, monocytes; CD15, neutrophils; CD19, B lymphocytes; CD235a, erythrocytes; CD4, T helper cells; CD56, natural killer cells; and CD8, cytotoxic T lymphocytes. **C** The miR-92a-3p targets of PfEMP1 reconfirmed by *RNAhybrid* tool, the plot representing the minimum free energy of miR-92a-3p with the target sequences (*X*-axis represents the PlasmoDB Ids for EMP1 sequences and *Y*-axis represents the minimum free energy). **D** Expression patterns of miR-142-3p, miR-15b-5p, miR-16-5p, miR-19b-3p, miR-223-3p, miR-451a, and miR-92a-3p during different time points of infection in malarial patients. *Y*-axis indicates the difference in infection status, and the *X*-axis indicates the expression of different miRNAs shown, the scale indicating expression in TPM. **E** In vitro expression of miR-92a-3p (transcript count) in red blood cell (RBC) and infected red blood cell (iRBC). Each dot represents a region of interest, and the significance was determined by Mann-Whitney *U* test

Red cells contribute significantly to the levels of whole blood miRNAs during malaria infection (Dieng et al. 2020). On analyzing the expression for the seven potential miRNAs in peripheral blood cells from blood miRNA datasets (Juzenas et al. 2017), we observed an enhanced expression for miR-451a, miR-92a-3p, and miR-16-5p while, moderate expression for miR-142-3p, miR-15b-5p, miR19b-3p, and miR-223-3p in the erythrocytes, thus confirming their presence in enucleated red cells (Fig. 4A, B). Predicted interactions obtained from miRanda were revalidated using RNA*hybrid* tool. The plot in Fig. 4C represents the minimum free energy of miR-92a-3p binding with multiple PfEMP1 target sequences showing the predictions.

The expression profiles for the miRNAs were analyzed from the data published by Mantel et al. (2016) and Dieng et al. (2020) and explored in clinical subtypes of infection (Mantel et al. 2016; Dieng et al. 2020). We observed a reduced expression for miR-92a-3p in the infected red blood cells during the symptomatic phase compared to that detected before infection (Fig. 4D, E). This is in line with our analysis where a drastic reduction in the expression level for miR-92a-3p was observed over time while other miRNAs exhibited dysregulated patterns over the course of infection.

### Integrated in silico analysis for miR-92a-3p

**Fig. 5:**
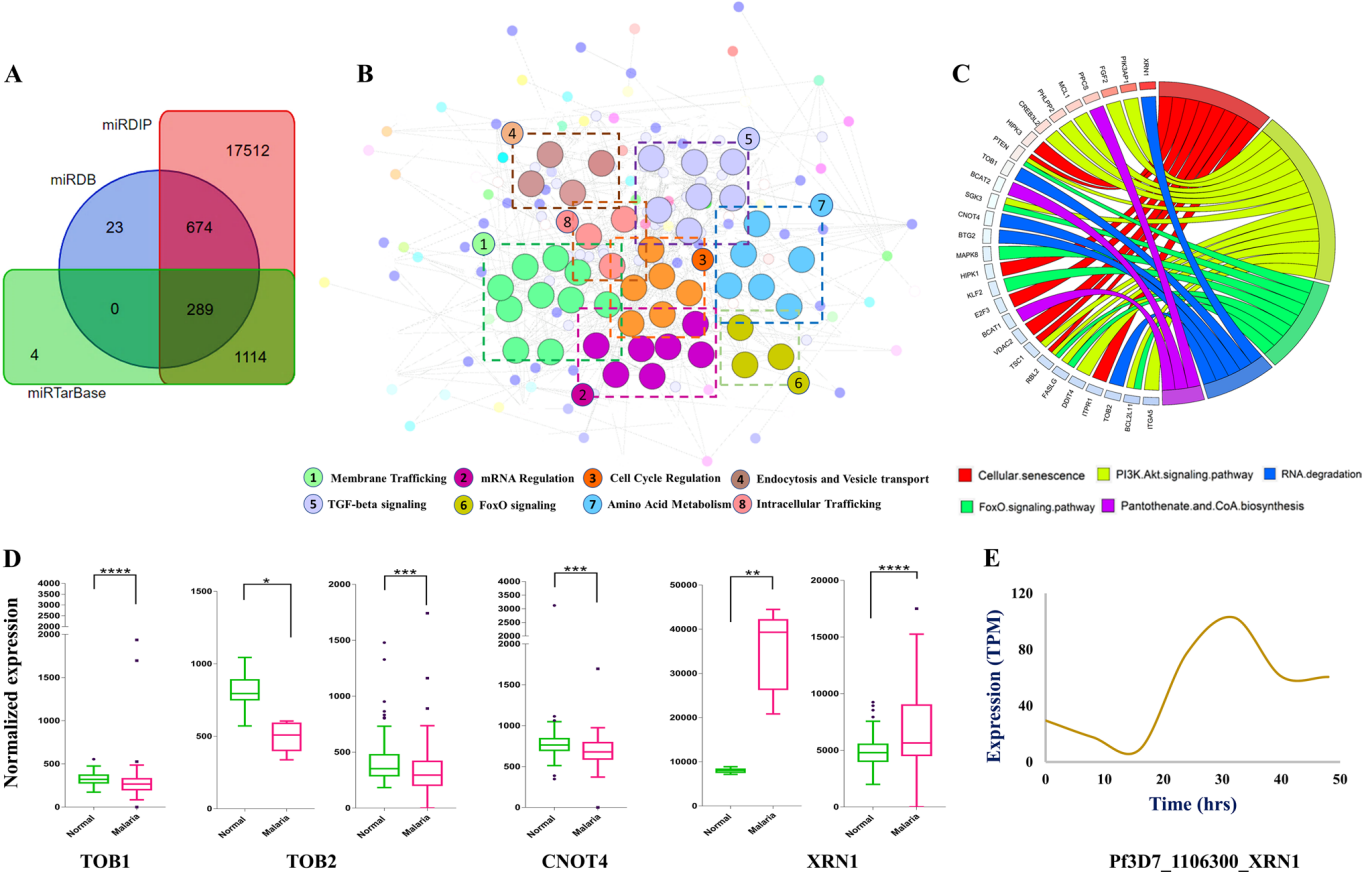
Integrative analysis for miR-92a-3p gene targets in human host. **A** Venn diagram representing 289 common human gene targets of miR-92a-3p identified from miRTarBase, miRDB, and miRDIP databases. **B** Network analysis of significant modules in protein-protein interaction generated using MCODE. **C** Representation of pathway analysis for target genes generated using GOPlot. **D** Expression analysis of significant genes involved in RNA degradation pathway in normal and malarial samples from GSE50957 and GSE52166 (GSE50957: *TOB1*, *BTG2 ns*, GSE52166: *CNOT4*, *BTG2 ns*). **E** Expression of *P. falciparum* XRN1 during the 48 h of infection cycle

Human host gene-targets of miR-92a-3p were identified from miRTarBase, miRDB, and miRDIP databases (Supplementary Table S5), and the 289 genes commonly found in the three (Fig. 5A) were considered for further analysis. The protein functional network included 289 nodes (genes) and 452 edges (interactions), with protein-protein interactions (PPI) enrichment *p* value 9.99e−16. MCODE clusters for eight significant modules (Supplementary Table S6) were identified from PPI networks and their enriched functions is illustrated in Fig. 5B. Pathway analysis for target genes were generated using GOPlot (Fig. 5C). The key genes belonged to pathways such as cellular senescence, PI3K/Akt signaling, RNA degradation, FoxO signaling, and pantothenate CoA biosynthesis. To analyze the contribution of human host in the miRNA degradation process, we explored the significant genes involved in RNA degradation pathway and compared their expression in normal and malarial samples (Mann-Whitney *U* test, *p*<0.05) obtained from GSE50957 and GSE52166. The gene *TOB1*, *TOB2*, and *CNOT4* showed a lower expression contrary to *XRN1* that exhibited an extremely increased expression in infected individuals (Fig. 5D). *XRN1*, a 5’-3’ exoribonuclease is well known for its role in RNA processing and degradation. To explore if the *P. falciparum* XRN1 (PfXRN1) play a defensive role by targeting human miRNAs for parasite survival, the transcripts (Pf3D7_1106300) from PlasmoDB were screened. The results showed an increased expression of PfXRN1 over time as presented in Fig. 5E. To assess the contribution of miR-451a, miR-16-5p, miR-142-3p, miR-15b-5p, miR19b-3p, and miR-223-3p in RNA degradation mechanisms, we checked for their human gene-targets and performed pathway analysis. However, no targets were identified for the genes indicating involvement of other RNA degradation pathways (Supplementary Table S5).

### Immunological imbalance during symptomatic malaria

**Fig. 6:**
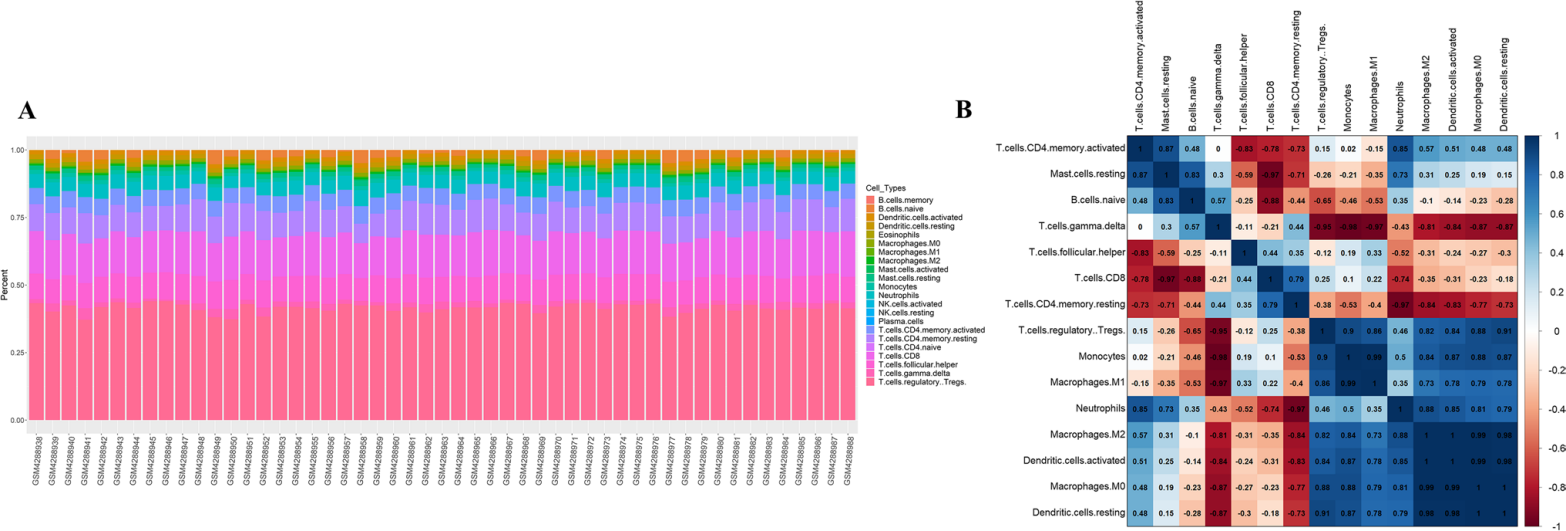
Immune infiltration analysis in symptomatic malaria. **A** The bar chart summarizes the percentage of infiltrating immune cells in malaria samples where each colour in the chart represents the type of immune cell. **B** Correlation analysis illustrating inverse correlation for most of the T cell types with other immune cells. The *X*- and *Y*-axis denotes the immune cell types, and the scale denotes the level of correlation between two cells (blue indicates positive correlation and red indicates negative correlation)

To determine the immune correlates when levels of miR-92a-3p decreased during symptomatic infection, we performed immune infiltration analysis in malarial patients (GSE144476). The CIBERSORT exploratory analysis generated the proportions of each immune cell types with fractions totalling to 100%. The results of the total proportion of infiltrating immune cells in malarial samples is presented in Fig. 6A. The correlation matrix obtained as shown in Fig. 6B presented a strong negative correlation of T cells subsets (resting CD4^+^ T cells, γδ T cells, CD8^+^ T cells) with other immune cell types such as monocytes, regulatory T cells, neutrophils, mast cells resting, and macrophages M1. The strongest negative correlation coefficient of −0.98 was obtained between gamma-delta T cells (γδ T) and monocytes, followed by; γδ T cells with macrophages M1, T cells CD8 with mast cells resting, and T cells CD4 memory resting with neutrophils with a correlation coefficient of −0.97.

### Implications of miR-92a-3p in symptomatic malaria

**Fig. 7:**
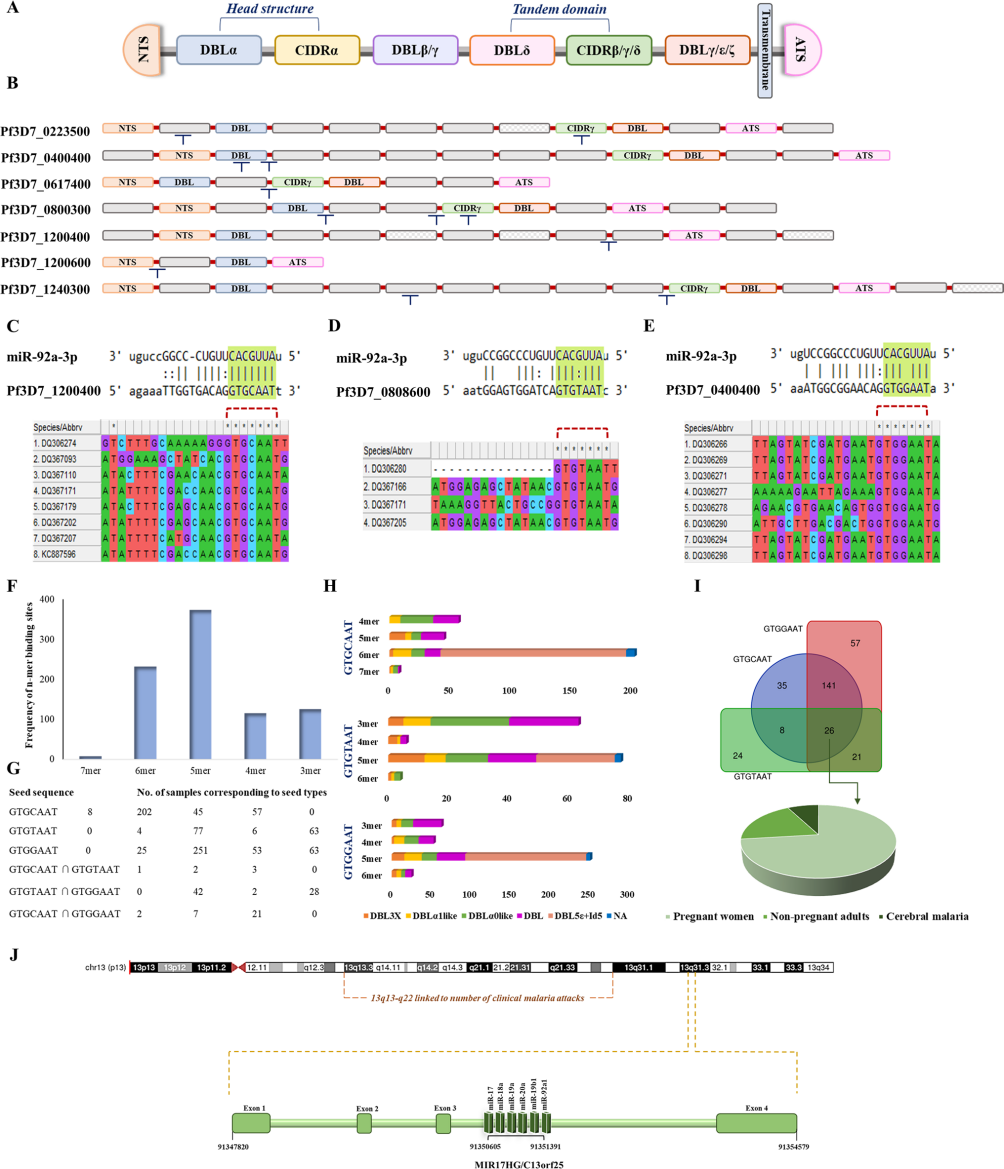
Genetic and epigenetic regulation during symptomatic malaria. **A** Genomic organization and domain classification for PfEMP protein: extracellular domain that consists of N terminal segment (NTS), varying combinations of duffy binding like (DBL) and cysteine rich interdomain regions (CIDR), a transmembrane domain followed by an intracellular acidic terminal segment (ATS). DBLα and CIDRγ domains were identified to be the targets for miR-92a-3p. **B** The binding motifs identified for the miR-92a-3p are shown. **C**, **D**, **E** Putative targets of miR-92a-3p on EMP sequences procured for the *P. falciparum* infected patient samples. **F**, **G** Patients categorized according to the seed sequence and frequency of n-mer binding sites with respect to seed types. **H** Number of DBL domains targeted by miR-92a-3p in accordance with the seed sequence and types. **I** Venn diagram representing 26 samples sharing the three possible miR-binding seed regions. The 26 samples were further categorized as per the clinical phenotypes and represented as a pie chart, where *n*=19 are pregnant women, *n*=5 are non-pregnant adults, and *n*=2 have cerebral malaria. **J** 13q13-q22 has previously been shown to be linked to number of *P. falciparum* malaria attacks. miR-92a-3p that belongs to C13orf25/ miR-17-92 cluster is located on human chromosome 13q31.3, suggesting epigenetic roles of non-coding RNAs for disease management

The miR-92a-3p was localized to band 31 on the long arm of chromosome 13 and belonged to miR-17-92 cluster as extracted from UCSC genome browser. The region on chromosome 13 (Fig. 7J) was also linked to clinical and parasitological malaria traits identified through genome wide linkage studies performed in two independent longitudinal cohort surveys in Senegal (Sakuntabhai et al. 2008).

The binding motifs for the miR-92a-3p in Pf3D7_EMP1 transcripts were identified to reside within the sequences those code for DBLα domains, disordered regions as well as CIDRγ domains (Fig. 7B), possibly enabling disruption of variants associated with clinical malaria. Additionally, out of 460 EMP1 coding sequences obtained for the *P. falciparum* infected patient samples, 8 patients manifested the most important canonical 7-mer miRNA seed type, “GTGCAAT” (Fig. 7C) followed by targets with wobble base-pairing (Fig. 7D, E and Supplementary Table S7). The patient samples available from PlasmoDB covered the regions corresponding to duffy binding like (DBL) domain subtypes viz. DBL3X, DBLα1 like, DBLα0 like, DBL5ε, and DBL in general. Patients were grouped and visualized in accordance with the n-mer seed match (Fig. 7F), where n-mers refer to the length of the complementary sequences, seed sequences (Fig. 7G) and the number of domain subtypes being targeted by the miRNA (Fig. 7H). The sequences for which the domain information was not available has been indicated as NA. Of note, all the samples exhibited sites compatible to miRNA binding with varying degree of specificity and efficacy. On analyzing the clinical phenotypes for the patients most susceptible to the miR-directed target degradation, possessing all the three possible seed complements (*n*=26), we observed that the majority of them are pregnant women (*n*=19), followed by non-pregnant adults (*n*=5), and patients with cerebral malaria (*n*=2) (Fig. 7I).

## Discussion

Human host erythrocytic miRNA-mediated regulation of *Plasmodium* genes and the clinical phenotypes of malaria have been established by evidences that have emerged during recent times (LaMonte et al. 2012; Rangel et al. 2019; Gupta et al. 2021b; Prabhu et al. 2021). Currently, we have performed in silico analysis for identifying the miRNA target sites of the *Plasmodium* genes that are involved in the intraerythrocytic developmental cycle. The transcriptomic datasets containing information on mRNA and miRNA expression levels in blood stage of infection at various time points were also analyzed. The expression profiles of the transcripts and their miRNAs demonstrated an increase in miRNA expression of parasitized erythrocytes leading to potential parasite gene regulation. The time course of expression profiles for miRNAs and their mRNA targets during infection demonstrates miR-19b-3p, miR-142-3p, miR-223-3p, miR-92a-3p, miR-15b-5p, miR-451a, and miR-16-5p contributing to modulation of the parasite gene expression.

Clinical studies have shown higher expression of miR-15b-5p detected in extracellular vesicles released in the plasma of *P. vivax* infected individuals (Ketprasit et al. 2020). The miRNAs were differentially expressed in parasite infected erythrocytes and the vesicles derived from them (Mantel et al. 2016). A better control of *Plasmodium* growth was also observed in individuals expressing higher levels of miR-92a-3p as a result of an increased frequency of T cells as an indicator of immune response to malaria (Burel et al. 2017). The patterns derived from our analysis also indicate a lower expression level for miR-92a-3p in infected RBCs. *Plasmodium* infection was shown to induce upregulation of miR-19b-3p, miR-223-3p, and miR-142-3p in in vivo models of cerebral malaria (Martin-Alonso et al. 2018). The virulent gene, PfEMP1, was shown to be targeted by multiple miRNAs, thereby magnifying the miRNA effect. miR-451a and miR-223-3p were considered to be the primary regulators of *P. falciparum* gene expression as evidenced by Grinev et al. (2021). The primary goal of this study is targeting the intraerythrocytic stage, the center-stage phase for vaccine targets. Interestingly, miR-92a-3p is found to be the regulator of the most virulent gene “PfEMP1,” a key factor contributing towards pathogenicity and immune evasion of *P. falciparum*.

MiRNA expression is controlled by a variety of factors and molecular processes, including those that impact DNA copy number, transcription factors, CpG methylation, and the availability of the miRNA-binding site in the transcript. Eukaryotic exoribonucleases like *XRN1* are well known for their role in a number of RNA processing activities, like mRNA and non-coding RNA degradation pathways, control mechanisms for aberrant tRNAs and mRNAs, and processing of intron lariats following splicing (Medina et al. 2014). *XRN1* was shown to target extracellular miR-223-3p, and its silencing slowed down the miRNA decay in the recipient cells (Zangari et al. 2017). Additionally, it has also been reported to regulate the expression of miR-277-3p in *Drosophila* and executed miR-382 decay in human cells (Jones et al. 2013). Besides, PfRNase II also appeared to modulate the expression of *var* genes in severe malaria (Zhang et al. 2014).

Consistent with these observations, we suppose a similar miRNA degradation mechanism being controlled either by human *XRN1* (*hXRN1*) or plasmodial XRN1 (pXRN1), in regulating miR-92a-3p levels during symptomatic malarial infection. Besides, gene expression analysis revealed a significantly higher expression of *XRN1* in infected individuals compared to the healthy controls along with a comparatively higher pXRN1 during the course of infection suggesting evolutionary fitness of the virulent EMP gene against the targeting miRNA. It is highly possible that to overcome the inhibitory effect of miR-92a-3p on the EMP1 genes, the pXRN1 activity is initiated that modulates the disease outcome in symptomatic cases as shown by our analysis.

Interestingly, the miRNA resides on chromosome 13 and belongs to the C13orf25/miR-17-92 cluster, that is under the transcriptional regulation by multiple factors including c-Myc, E2F, etc. c-Myc was reported to be downregulated in uncomplicated malaria initially but returned to its normal expression levels with clinical recovery (Colborn et al. 2015). Despite being co-transcribed, individual miRNAs of this cluster are processed with varying efficiencies or they are occupied already with their cognate binding sites and may operate independently to regulate multiple signaling cascade as has been previously reported (Landskroner-Eiger et al. 2015). By preventing equal accessibility of the pre-miR domains to the enzymatic cleavage, the compact tertiary structure of pri-miR-17-92a has been documented to regulate distinct expression of the component miRNAs (Chaulk et al. 2011; Chakraborty et al. 2012). Though there were differences in the expression patterns of other members of the miR-17-92 cluster (hsa-miR-17, hsa-miR-18a, hsa-miR-19a, hsa-miR-19b-1, hsa-miR-20a) during *Plasmodium* infection; however, their levels were lower in uninfected erythrocytes (Supplementary Table S4). Besides, among the cluster members, hsa-miR-92a-3p showed higher binding efficiency as well as lower Gibbs energy. Genetic studies have demonstrated an association of 13q13-q22, 5q31-q33, 10p15, and 1p36 with the risks of clinical malaria caused by *P. falciparum* (Rihet et al. 1998; Flori et al. 2003; Sakuntabhai et al. 2008). Also, regions on chromosomes 9, 5, 17, 11, and 3 have been linked to parasite density in *P. chabaudi* infection model (Timmann et al. 2007). Here, we present an evidence of plausible epigenetic disease regulation being controlled by non-coding RNAs at 13q31.3.

*P. falciparum* has evolved to subvert acquisition of the protective immune responses through the expression of variant surface antigens (VSAs) on the infected red blood cell membranes, facilitating their sequestration. The major VSA, PfEMP1 which is responsible for rosetting and cell adhesion during early phases of infection is highly expressed and the protein encoding PfEMP1 gene is present in about 60 copies in all the 14 chromosomes (Nortey et al. 2022). The gene comprises of two exons where exon I codes for the extracellular and transmembrane portion and exon II codes for the intracellular domains. But then N-terminal region which consists of different domains (Duffy binding like (DBL)- α, β, γ, δ, ε, ζ, and cysteine-rich interdomain region (CIDR)- α, β, γ, δ) is highly variable and C-terminal domain consists of acid rich terminal sequence that is highly conserved among all the copies (Fig. 7A). Each parasite expresses a restricted subset of PfEMP1 genes in multiple phenotypes associated with severe malaria, asymptomatic malaria, and uncomplicated malaria, although switching its expression at each reinvasion cycle (Jensen et al. 2004). These variations result in distinct forms of PfEMP, thus accounting for disparities in their host binding capabilities. Variants encoding DBLα0-CIDRα2-6, DBLα1-CIDRα1, DBLα1-CIDRβ/γ/δ, DBLβ subtypes, and DBL3x domains have shown to be associated with cluster determinant 36 (CD36), endothelial protein C receptor (EPCR), complement receptor 1 (CR1), intercellular adhesion molecule 1 (ICAM1), and chondroitin sulphate A (CSA) binding, respectively, facilitating pathogenesis of severe malaria (Tessema et al. 2019; Obeng-Adjei et al. 2020).

The binding sites for the miR-92a-3p were identified to reside within the sequences that code for DBLα domains, disordered regions, and CIDRγ domains in the *Pf3D7* transcripts. Similarly, all the patient samples for the PfEMP1 gene exhibited compatibility towards targeted miRNA degradation. The sequences covered the regions corresponding to the duffy binding like (DBL) domain subtypes viz*.* DBL3X, DBLα1 like, DBLα0 like, DBL5ε, and DBL in general. Interaction of DBL domains with several host receptors facilitate parasite sequestration and rosette formation influencing disease severity. The DBL3X domain is believed to mediate placental infections by binding to the CSA receptors, while the DBLα and DBL5ε domains bind CR1, heparan sulphate, and IgM, respectively (Duffy et al. 2014). Multivalency and high levels of glycosylation of IgM has been demonstrated to promote contact and enhance the binding affinity of the cells forming rosette (Clough et al. 1998). Additionally, categorization of samples as per the clinical phenotypes revealed a diversity in clinical presentations such as cerebral malaria, placental malaria, and uncomplicated malaria.

Although PfEMP might be considered as an unlikely candidate for vaccine targets due to their extreme diversity, there is a strong rationale for developing vaccines that prevent disease severity. High-throughput approaches are required to identify the most significant PfEMP1 variants among the thousands circulating in the parasite populations. Consistent with the fact that PfEMPs are the primary contributors of complicated malaria and that targeting them is an ultimate challenge, we hypothesize that the development of miRNA mimics as potential therapeutic options. Taken together, this study supports the development of miR-92a-3p mimics as leading candidates conferring protection against clinical *P. falciparum* malaria.

*Plasmodium* infection triggers an array of complex host immune responses. The miR-17-92 cluster was identified to be critical for fidelity of follicular helper T cell differentiation, gene-expression, and regulating T cell-dependent responses. miR-17-92 cluster and miR-92a in general have also been reported to be involved in the imbalance of T cell subsets during infection. Following this, the immune infiltrates and the correlation matrix obtained (Fig. 6A, B) illustrated modulation of immune cells where T cells exhibited an inverse correlation with macrophages, neutrophils, and mast cells indicating immune dysfunction (Baumjohann et al. 2013; Khan et al. 2013; Kroesen et al. 2015; Liang et al. 2016; Kuo et al. 2019; Fujiwara et al. 2022). We observed a strong negative correlation of T cells in symptomatic malaria patients when miR-92a levels also decreased suggesting a crucial role of this cluster in managing malaria.

### Limitations

An important limitation of this study is the paucity of erythrocyte-specific miRNA profiles of malaria-infected individuals. Nevertheless, our analysis could be further subjected to the required validations. The results contribute to identification of several candidate targets for interventional strategies in the erythrocytic stage of the parasites. We identified a majority of mRNAs being targeted encode, the most virulent gene PfEMP1. The findings allow us to consider the phenomenon of antiparasitic defensive strategy exhibited by the human host and further experimental verification can identify the most suitable miRNAs for targeted interventional therapies. miRNA profiles of malaria patients with different clinical infections and the levels of plasmodial genes can be monitored to follow the mechanisms determining the outcome of infection. The vesicle-mediated miRNA delivery mechanisms add on another layer to this development.

### Future perspectives

A study of the human host miRNAs expressed in liver cells during parasite infection were not encountered in the literature. Such studies would offer evidence as to the host mechanisms favouring infection/resistance. Attempts are being made using antimiRs and miRNA mimics to treat different diseases therapeutically. These include mimics of miR-34 and antimiRs targeted towards miR-122 that have advanced to phase I and phase II clinical trials for treating cancer and hepatitis respectively (Rupaimoole and Slack 2017). Similarly, phase I and phase II clinical studies are being conducted to evaluate the efficacy of miR-21, miR-29, miR-92, miR-16, and miR-155-based therapies in treating cancer, heart failure, wound healing, and other disorders (Gupta and Wassmer 2021; Schwendt et al. 2019). Research into the role of miR-92a-3p mimics in regulating the virulent genes and processes pertinent to symptomatic malaria may be promising.

## Supplementary information

ESM 1:

## Data Availability

All data generated or analyzed during this study are included in this article and its supplementary information files.

## Abbreviations

ATS: Acidic terminal segment
C13orf25: Chromosome 13 open-reading frame 25
CD36: Cluster determinant 36
CIDR: Cysteine-rich interdomain region
CNOT4: CCR4-NOT transcription complex subunit 4
CR1: Complement receptor 1
CSA: Chondroitin sulphate A
DAVID: Database for annotation, visualization, and integrated discovery
DBL: Duffy binding like
ENA: European nucleotide archive
EPCR: Endothelial protein C receptor
G6PD: Glucose-6-phosphate dehydrogenase
GEO: Gene expression omnibus
GOEA: Gene ontology enrichment analysis
HLA: Human leukocyte antigen
ICAM1: Intercellular adhesion molecule 1
MCODE: Molecular complex detection
miRNAs: MicroRNAs
NTS: N terminal segment
*Pf*: *Plasmodium falciparum*
EMP1: Erythrocyte membrane protein 1
PPI: Protein-protein interactions
γδ T: Gamma. delta T cells
TPM: Transcripts per million
TOB: Transducer of ERBB2
VSA: Variant surface antigen
WHO: World Health Organization
XRN1: Exoribonuclease 1
